# Phenomenological Incorporation of Nonlinear Dendritic Integration Using Integrate-and-Fire Neuronal Frameworks

**DOI:** 10.1371/journal.pone.0053508

**Published:** 2013-01-07

**Authors:** Douglas Zhou, Songting Li, Xiao-hui Zhang, David Cai

**Affiliations:** 1 Department of Mathematics, MOE-LSC, and Institute of Natural Sciences, Shanghai Jiao Tong University, Shanghai, China; 2 Institute of Neuroscience and State Key Laboratory of Neuroscience, Shanghai Institutes for Biological Sciences, Chinese Academy of Sciences, Shanghai, China; 3 Courant Institute of Mathematical Sciences and Center for Neural Science, New York University, New York, New York, United States of America; McGill University, Canada

## Abstract

It has been discovered recently in experiments that the dendritic integration of excitatory glutamatergic inputs and inhibitory GABAergic inputs in hippocampus CA1 pyramidal neurons obeys a simple arithmetic rule as 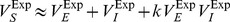, where 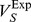, 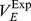 and 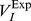 are the respective voltage values of the summed somatic potential, the excitatory postsynaptic potential (EPSP) and the inhibitory postsynaptic potential measured at the time when the EPSP reaches its peak value. Moreover, the shunting coefficient 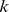 in this rule only depends on the spatial location but not the amplitude of the excitatory or inhibitory input on the dendrite. In this work, we address the theoretical issue of how much the above dendritic integration rule can be accounted for using subthreshold membrane potential dynamics in the soma as characterized by the conductance-based integrate-and-fire (I&F) model. Then, we propose a simple I&F neuron model that incorporates the spatial dependence of the shunting coefficient 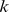 by a phenomenological parametrization. Our analytical and numerical results show that this dendritic-integration-rule-based I&F (DIF) model is able to capture many experimental observations and it also yields predictions that can be used to verify the validity of the DIF model experimentally. In addition, the DIF model incorporates the dendritic integration effects dynamically and is applicable to more general situations than those in experiments in which excitatory and inhibitory inputs occur simultaneously in time. Finally, we generalize the DIF neuronal model to incorporate multiple inputs and obtain a similar dendritic integration rule that is consistent with the results obtained by using a realistic neuronal model with multiple compartments. This generalized DIF model can potentially be used to study network dynamics that may involve effects arising from dendritic integrations.

## Introduction

A single neuron may receive and integrate thousands of excitatory and inhibitory synaptic inputs from its dendritic tree. Through spatial and temporal integration of these synaptic inputs, neurons in the cortex process information efficiently and produce output signals known as spike trains. In order to understand how the brain works, it is important to understand the rules that govern dendritic integration. However these rules remain to be fully elucidated. For the integration of multiple excitatory inputs, cable theory [1], [2] was developed and was successfully applied to describe passive properties of dendrites as observed in experiments [3], [4]. In order to consider active properties of dendrites such as the activity of some voltage-gated ion channels and the occurrence of dendritic spikes [5]–[7], a two-layer network model was proposed [8], [9]. This model has been supported by some experiments using focal synaptic stimulation and glutamate uncaging [10], [11]. However, as reviewed in Refs. [12], [13], there are still many other important properties of dendritic integration, such as spike timing, that cannot be captured by this model.

In contrast to theoretical and experimental results for the integration rule of multiple excitatory inputs as mentioned above, we know even less about the integration rule when both excitatory and inhibitory inputs are presented together. In order to describe dynamics of neuronal circuitry within the brain, it is important to understand how excitatory and inhibitory inputs are integrated. For example, the interactions of excitatory and inhibitory synaptic currents have been found to play an important role in many sensory systems [14]–[16]. Shunting inhibition is found to be able to control the gain of a neuron in the presence of excitatory synaptic inputs [17]. Inhibition can also modulate the frequency [18] and improve the robustness [19] of gamma oscillations through nonlinear interactions with synaptic excitation. Therefore, it is important to determine precise rules that govern synaptic excitation-inhibition integration in order to achieve a good understanding of underlying computational mechanisms for these neurophysiological phenomena.

**Figure 1:**
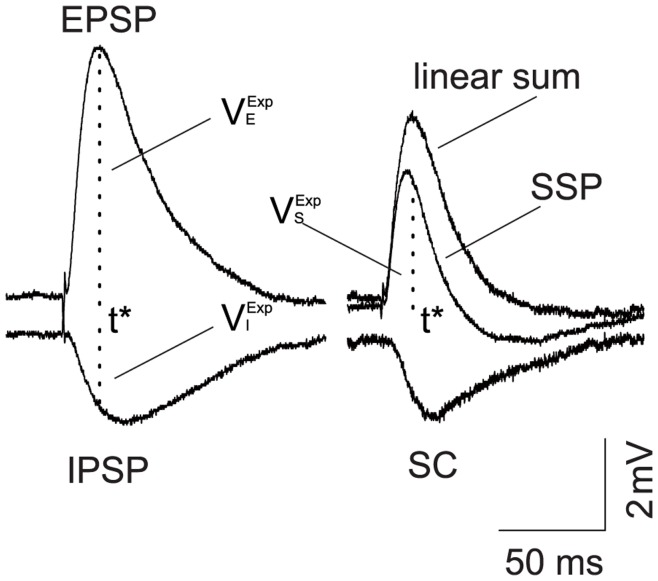
Experimental measurement of EPSP, IPSP and SSP. The time when EPSP reaches its peak value is denoted by 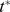. 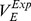 and 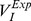 represent the amplitude of EPSP and IPSP at time 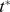, respectively. 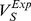 represents the amplitude of SSP at time 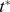. SC is the difference between the SSP and the linear sum of the individual EPSP and IPSP measured under separate excitatory and inhibitory inputs. When the excitatory input and the inhibitory input are elicited simultaneously, the response amplitude measured at the soma is found to be smaller than that of the linear sum. (modified from Ref. [20]).

**Figure 2:**
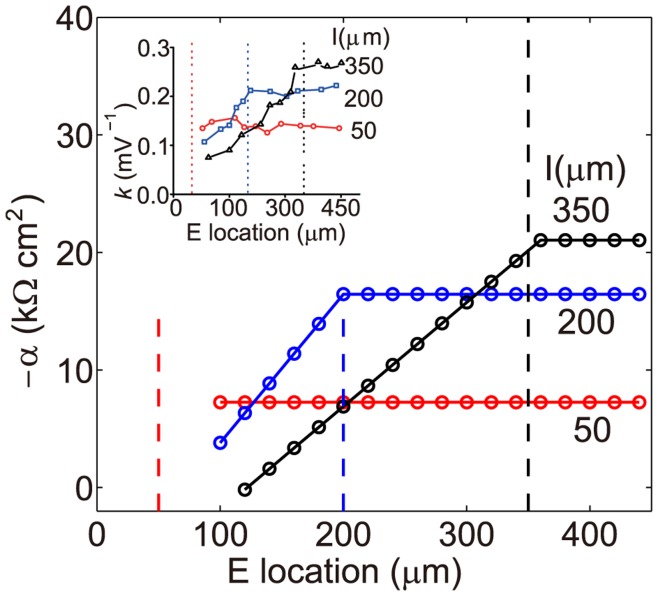
The spatial dependence of 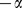 obtained from Eq. (9). Given three fixed inhibitory input locations, we can parameterize 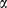 in Eq. (9) by identifying 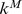 with the shunting coefficient measured in the experiment [20] to obtain the spatial dependence of 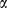. Inset: the experimental measurements of the shunting coefficient [20] as a function of the distance between the excitatory input site and the soma for three fixed inhibitory input sites on the dendrite (marked by dashed lines).

Recently, a quantitative description of a dendritic integration rule has been uncovered in experimental results from CA1 pyramidal neurons in one of our authors' lab [20]. In the experiment, when the excitatory glutamatergic input and the inhibitory GABAergic input were elicited simultaneously with two iontophoretic pipettes at adjacent locations on a dendritic trunk, the response measured in the soma was found to be always smaller than the linear sum of the individual excitatory postsynaptic potential (EPSP) and inhibitory postsynaptic potential (IPSP) measured in the soma separately as shown in Fig. 1. In Fig. 1, 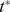 denotes the time when the EPSP reaches its peak value, denoted as 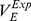, which is referred to as the amplitude of EPSP. The values of IPSP and the summed somatic potential (SSP) at time 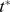 were denoted by 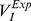 and 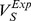, referred to as the amplitudes of IPSP and SSP, respectively. The arithmetic summation rule for the dendritic integration in Ref. [20] can now be expressed as
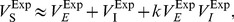
(1)where the third term on the right-hand side of Eq. (1) is the so-called shunting component (SC) with 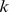 as the shunting coefficient [20]. Such a relationship was also found for the mean values of EPSP, IPSP and SSP, instead of the voltage values at time 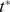 [20]. In addition, the shunting coefficient 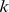 depends on locations of both the excitatory and the inhibitory stimulus but not the amplitudes of EPSP and IPSP. As shown in the inset of Fig. 2, when the location of the inhibitory input on the dendritic trunk is fixed and the excitatory input is located in between the soma and the inhibitory input site, 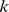 increases as the distance between the excitatory input and the soma increases. On the other hand, when the excitatory input is located further away from the soma than the inhibitory input site, 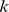 remains almost constant with further increases in the distance between the excitatory input site and the soma.

In Ref. [20], numerical simulation based on the software NEURON [21] was also performed to examine this integration rule. The simulation used a reconstructed spatial structure of the CA1 neuron, which included 200 compartments, four different ion channels, and four different neurotransmitter receptors. These components are used to mimic both the active channel properties and passive cable properties of the dendrite of the real neuron. The simulation was able to account for many aspects of the experimental results. However, the value of 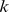 produced by the simulation was approximately only one half of the experimentally measured value, indicating that the constructed multi-compartmental neuron is still not able to capture quantitatively the effects of dendritic integration of a real neuron. Moreover, there are some other experimental results as reported in the supplementary text of Ref. [20] that have not been addressed by this multi-compartmental model, such as the case when excitatory inputs and inhibitory inputs are no longer synchronous–-clearly, this situation often occurs and excitatory and inhibitory stimulations may not always occur at precisely exactly the same time as in Ref. [20]. (Of course, excitatory and inhibitory events can be often highly correlated in time [22].) We further note that the model used in simulations contains many compartments and parameters, rendering it difficult to study analytically. In addition, there are other theoretical issues that need to be clarified. For example, it is not clear how the nonlinear SC term arises mechanistically by using this simulation approach. Furthermore, neuronal coding often involves dynamics of networks. However, it would be difficult to implement this multi-compartmental model in network simulation in that the complexity of such a neuron model would make the computational cost impractical. Therefore, it is desirable to incorporate the dendritic integration features into a simpler neuronal model that has the potential to address these theoretical issues.

Many spiking neuronal models have been developed to capture spike dynamics of real neurons [23]–[30]. Each model has its own advantages and disadvantages with respect to the understanding of neuronal spiking dynamics [31]. Since the data measured in the experiment [20] were all collected at the *soma*, we first address the issue of whether we can understand this dendritic integration rule through somatic properties of a single-compartment point-neuron model. Considering the trade-off between biological plausibility and theoretical complexity among those existing neuronal models [31]–[34], we choose the conductance-based integrate-and-fire (I&F) model as the basic model for the investigation of the dendritic integration rule. Note that the voltage traces measured in the experiment [20] involved only subthreshold dynamics. The I&F model is well-suited to investigate the dendritic integration rule as models of the I&F type have been experimentally shown to quantitatively capture the subthreshold dynamics of neurons [34], [35]. Surprisingly, this simple model can produce the arithmetic rule [Eq. (1)] for the case in which the stimulus location is *fixed*. Therefore, it suggests that somatic membrane potential dynamics may play a role in the so-called dendritic integration rule. Through our theoretical analysis, we demonstrate that the nonlinear SC term arises from the multiplication of conductance and voltage in the synaptic input of the neuron. We further point out that, in a static two-port analysis, the product between excitatory and inhibitory conductances gives rise to the product between EPSP and IPSP. Using the insight derived from these analyses, finally, we develop a dendritic-integration-rule-based I&F (DIF) model to phenomenologically incorporate the spatial effect of the integration. This phenomenological model can not only incorporate the dendritic integration rule, but also capture experimental observations that have not been addressed by the multi-compartmental model [20]. In addition, it also yields predictions that can be further used to validate this model experimentally. We note that the original dendritic integration rule [Eq. (1)] is limited to the situation in which the excitatory and inhibitory inputs are concurrent. When excitatory and inhibitory inputs are not concurrent, the shunting coefficient 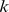 has to be measured again in experiments to obtain the corresponding dendritic integration formula. However, we show that the DIF model has extended the dendritic integration rule to dynamical situations and it can be used to study neuronal responses to any stimulus pattern with both excitatory and inhibitory inputs. Finally, we generalize the DIF model for studying the effect of multiple inputs and obtain related results that are consistent with those obtained using the NEURON software as reported in Ref. [20]. Because our model is an I&F type, clearly, it can easily be implemented in network dynamics to study certain effects arising from dendritic integrations.

The paper is organized as follows, we first show both theoretically and numerically that the arithmetic dendritic integration rule can partially be explained by somatic subthreshold membrane potential dynamics of the conductance-based I&F model. Next, using the insight derived from a static two-port analysis, we develop a simple DIF model to phenomenologically parameterize the spatial dependence of the shunting coefficient 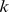. Our numerical results show that this model captures many neurophysiological phenomena as observed in experiments. Here, we further extend the DIF model to account for multiple inputs. In *Discussion*, we determine the parameter range and describe some predictions of the DIF model. In *Methods*, we introduce the conductance-based I&F model and present both analytical and approximate solutions to the I&F-type models. Here, we also recapitulate the static two-port analysis in detail.

## Results

### Subthreshold Membrane Potential in the Soma

**Figure 3:**
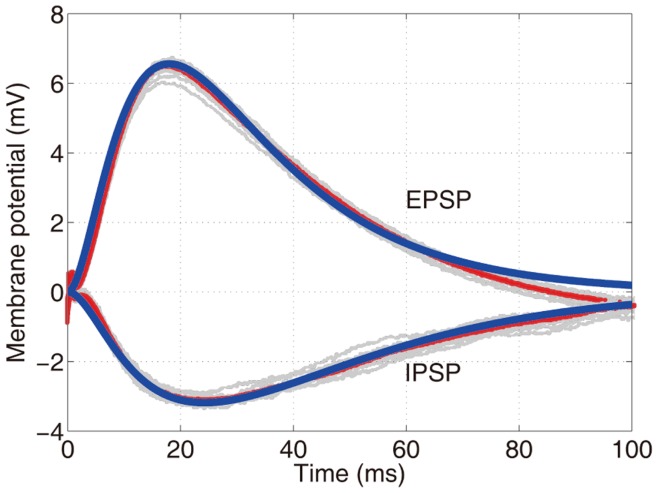
Reproduced profiles of EPSP and IPSP by the I&F model. The thick dark (blue online) lines are produced from the I&F model, the light gray lines represent EPSP and IPSP measured in the experiment for different trials, and the thin dark (red online) lines represent the trial-averaged responses in the experiment. Parameters in the I&F model are chosen as follows, 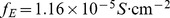, 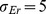
ms, 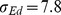 ms, 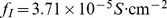, 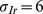 ms, and 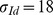 ms. (See *Methods* for details).

**Figure 4:**
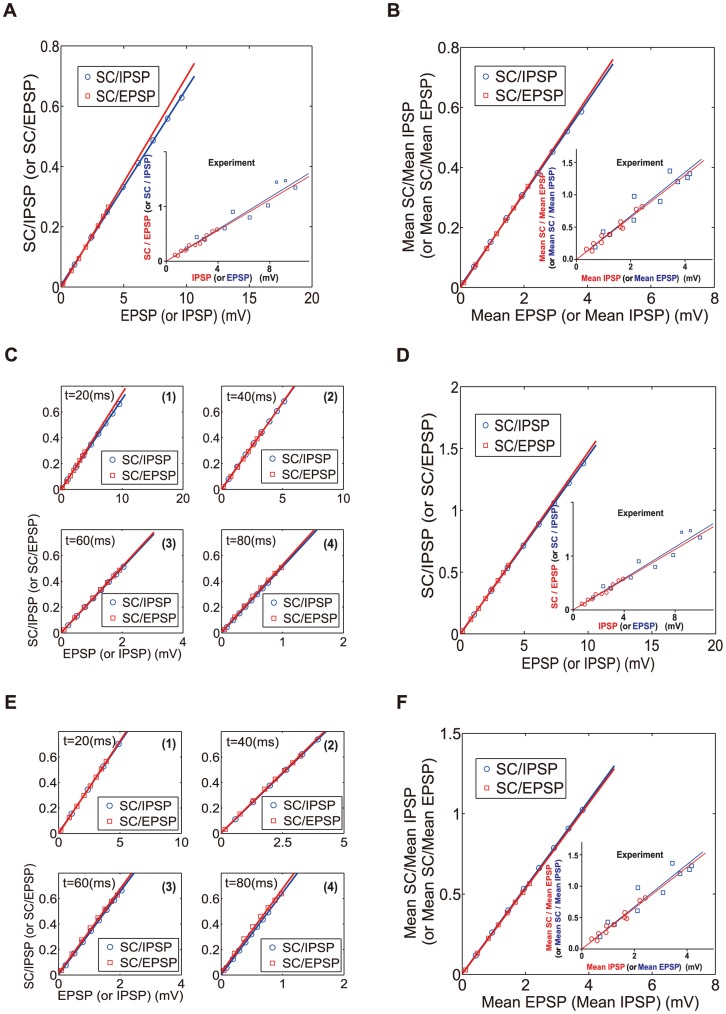
Dendritic integration rule obtained from the I&F model (A–C) and the modified I&F model (D–F). We choose the values of 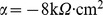 and 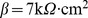 so that the value of 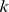 computed from Eq. (8) matches the value measured in the experiment [20]. Lines in figures indicate linear fits with slope 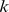. (A) Ratio between SC and EPSP (SC/EPSP) plotted against IPSP (red square online) and SC/IPSP plotted against EPSP (blue circle online) at time 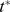 for the I&F model (Red online: 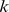 = 0.070; blue online: 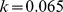). Inset: experimental measurement (Red online: 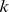 = 0.142; blue online: 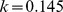). (B) Ratio between the mean SC and the mean EPSP (SC/EPSP) plotted against the mean IPSP (red square online), the mean SC and the mean IPSP (SC/IPSP) plotted against the mean EPSP (blue circle online) for the I&F model. Inset: experimental measurement. (C) the same as (A) but at time (1) 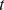 = 20 ms, (2) 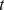 = 40 ms, (3) 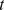 = 60 ms and (4) 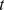 = 80 ms, respectively, as marked on the figures. (D) the same as (A) but for the modified I&F model (Red online: 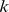 = 0.147; blue online: 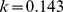). Inset: the same as the inset of (A). (E) the same as (C) but for the modified I&F model. (F) the same as (B) but for the modified I&F model. Inset: the same as the inset of (B).

It has been demonstrated that the I&F model can capture very well the subthreshold membrane potential dynamics in the soma of a real neuron when its membrane potential is below 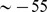 mV [34], [35]. The membrane potential used in the experiment [20] for determining the dendritic integration rule is precisely in this subthreshold regime. A natural question arises, that is, how much the dendritic integration rule can be accounted for by the somatic membrane dynamics. Here, we employ the conductance-based I&F model (see *Methods*) to address this question. First, we use the experimental data to determine appropriate parameters to reproduce the same profiles of EPSP and IPSP as measured in the experiment [20] (Fig. 3, see *Methods* for details). Then, we use a fourth-order Runge-Kutta method to solve the I&F model numerically. We denote the EPSP by 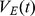, IPSP by 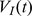, and SSP by 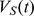. The time 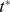 is the time when 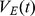 reaches its peak value. The values of 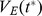, 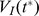 and 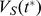 are referred to as the amplitudes of EPSP, IPSP and SSP, respectively. In order to verify the product form of the SC term, we follow the same data processing procedure as in Ref. [20]: by setting the EPSP amplitude at a fixed value while varying the IPSP amplitude, we examine whether the SC amplitude linearly depends on the IPSP amplitude. Conversely, for a fixed amplitude of IPSP, we examine whether the SC amplitude linearly depends on the EPSP amplitude. As shown in Fig. 4A, the SC increases linearly with respect to the IPSP amplitude when the EPSP amplitude is fixed, whereas the SC increases linearly with respect to the EPSP amplitude when the IPSP amplitude is fixed. In addition, we observe that both straight lines in Fig. 4A have almost identical slopes and this relationship is exactly the same as the one observed in the experiment [20]. If we use the mean values 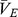, 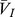 and 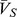 of EPSP, IPSP and SSP respectively, averaged over a time interval of 100 ms, the summation rule 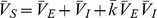 also holds as shown in Fig. 4B. This is also consistent with the experimental observations [20]. Our numerical results further show that the rule holds for 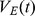, 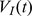 and 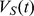 at any moment of time 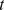 (i.e., not restricted to 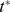) as demonstrated in Fig. 4C (1–4) for a selected set of times.

As shown above, the I&F dynamics of somatic membrane potential can exhibit the dendritic integration rule [Eq. (1)]. However, one may ask why this linear dynamics of somatic subthreshold membrane potential as described by the I&F model can produce such a nonlinear integration rule. Below, we answer this question analytically (see *Methods* for details). From Eq. (1), we have
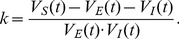
(2)


If we can demonstrate that 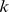 is independent of the amplitude of inputs, then the dendritic integration rule holds. Performing a Taylor expansion of the analytical solution to the conductance-based I&F model, we can obtain approximations of EPSP, IPSP and SSP (See *Methods*). Substituting these voltage values [Eqs. (22b), (23b) and (24b)] into Eq. (2), we can obtain an approximate 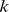, denoted by 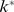 as
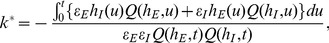
(3)where 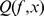 is a functional defined as 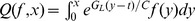. 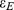 and 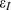 are the excitatory and inhibitory reversal potentials, respectively. 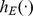 and 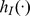 as given by Eqs. (17) and (18) only determine the profiles (rise and decay time scale) of EPSP and IPSP, whereas the excitatory and inhibitory inputs strength 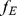 and 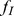 corresponding to the amplitudes of EPSP and IPSP as in Eqs. (15) and (16), do not appear in the expression of 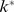 [Eq. (3)]. Therefore, 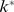 is independent of the amplitudes of EPSP and IPSP for any times.

For the mean EPSP and the mean IPSP, we can simply take integrals of 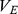, 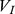, 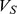 over the time interval 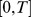 to obtain the mean values (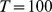
ms in the experiment [20]), where the mean is defined as 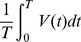. By definition, the shunting coefficient for the mean case can be evaluated as
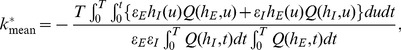
(4)which shows that 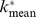 is independent of the EPSP and IPSP amplitudes for the same reason as mentioned in the analysis of Eq. (3). Therefore, the dendritic integration rule holds for the somatic membrane potential as modeled by the I&F dynamics. Through the above analysis, it can be seen that the nonlinearity in the dentritic integration rule ultimately arises from the product term of the conductance 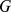 and the voltage 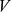 in the input current of the I&F neuron (see *Methods*).

### The DIF Neuronal Model

As is well known, the conductance-based I&F model is used to describe the membrane potential dynamics in the soma without taking into account dendritic structures. From the above analysis, it seems that the subthreshold membrane potential dynamics in the soma is able to explain the dendritic integration rule [Eq. (1)]. However, as can be seen in Fig. 4A, there is a difference of a factor of two between the value of the shunting coefficient 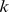 measured in the experiment and that of 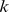 computed by the I&F model. Incidentally, the experimentally measured 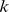 and the value obtained by the NEURON software in Ref. [20] also differ almost by a factor of two. Importantly, as observed in the experiment [20], the value of 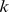 depends on the distance between the input sites and the soma. However, from Eq. (3), it can be seen that 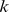 does not contain any spatial information explicitly. In the following, we will construct a simple phenomenological neuron model that incorporates the spatial dependence in the dendritic integration rule. As can be seen below, our new neuron model can account for additional observed experimental phenomena and will also be useful for network simulations that take into account effects arising from dendritic integrations.

To incorporate the spatial dependence of the dendritic integration rule, we first motivate the construction of our neuron model by a static two-port analysis [33] (see *Methods* for details). For the time-independent case, the conductance inputs of both excitation 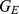 and inhibition 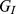 are constant, the transfer resistance between any two sites on the dendritic tree is also constant. Therefore, we can express the excitatory and inhibitory currents as 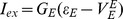 and 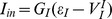, respectively. Here 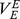 represents the membrane potential at the site of excitation and 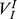 the membrane potential at the site of inhibition. We can obtain the membrane potentials 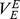 and 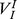 as 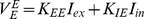 and 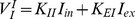, respectively [33]. Here 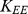 and 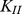 are local transfer resistances at the sites of excitation and inhibition, respectively. 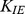 corresponds to the transfer resistance from the inhibitory site to the excitatory site and vice versa for 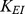. The membrane potential at the soma can be obtained by adding the excitatory and inhibitory contributions, i.e., 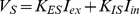. If the conductance inputs are sufficiently small, we can obtain the following relationship under the simultaneous drive
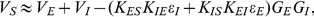
(5)where 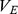 (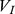) is the EPSP (IPSP) obtained with only excitatory (inhibitory) input, and 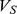 is the SSP in this static case. From *Methods*, we have that the product of 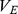 and 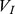 can be approximated by



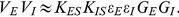
(6)Therefore, we can obtain Eq. (39), which has exactly the same form as the dendritic integration rule with a shunting coefficient 
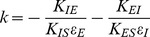
. We emphasize that this dendritic integration rule obtained through the two-port analysis is only valid for the static case. Clearly, we need to address the time-dependent case as in the experimental setup [20]. However, this static analysis provides us with an observation about possible mechanisms underlying the dendritic integration rule. Note that, in Eqs. (5) and (6), the shunting component encompasses the product term between the excitatory conductance 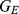 and the inhibitory conductance 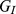, in turn, yielding the product, 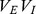, in Eq. (39). Therefore, the product, 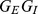, is the intrinsic origin of the shunting component in the static case [Eq. (38)]. Using this observation, we propose to generalize the I&F dynamics by introducing terms of 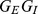 and obtain the following governing equation for a neuron:
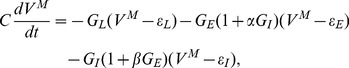
(7)where 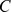 is the membrane capacitance, 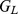 is the leaky conductance, 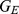 and 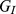 are the excitatory and inhibitory conductances, respectively. 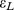 is the resting potential, 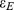 and 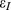 are the excitatory and inhibitory reversal potentials, respectively. In Eq. (7), for clarity of later discussions, we denote the membrane potential by 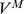. The parameters 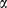 and 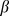 are used to parameterize the spatial effects of dendritic integration. In the following, we will refer to Eq. (7) as the modified I&F model.

First, we discuss how to determine the parametrization of 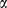 and 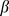. Considering the time-independent case, we denote individual EPSP and IPSP at the soma by 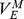 and 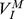, the SSP by 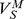. The condition 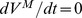 leads to 
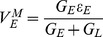
, 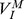 = 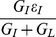 and 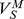 = 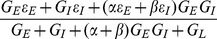. By imposing the condition that the dendritic integration rule holds, i.e., 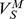 = 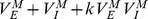, we can conclude that the parameters 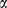 and 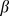 must satisfy 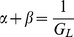 and 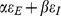 = 
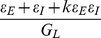
, from which, we obtain 
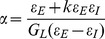
 and 
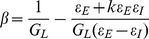
. Note that, in the limit of 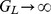, we have 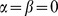, and the modified I&F model reduces to the standard I&F model. That is, the modified I&F model is naturally a generalized I&F model. We will show below that 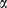, 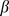 can indeed be used to parameterize the dendritic integration rule in a dynamical situation.

We now turn to the discussion that in the modified I&F model the dendritic integration rule holds for any moment of time, including the time when the EPSP reaches its peak. Solving the modified I&F model numerically using the fourth-order Runge-Kutta method, we have the relationship among 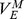, 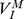, and 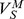 as Eq. (1) at the time when 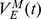 reaches its peak (Fig. 4D) as well as at any other times (Fig. 4E(1–4)). As shown in Fig. 4F, this integration rule is also valid if the mean values of EPSP, IPSP and SSP are used.

**Figure 5:**
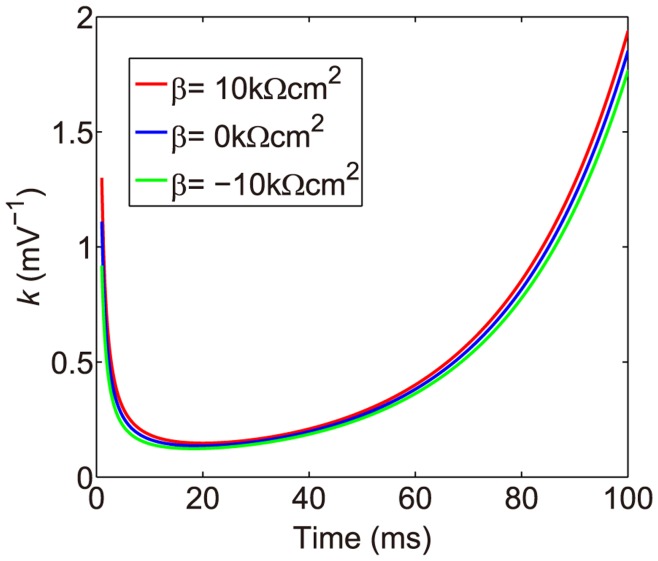
The shunting coefficient 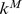 as a function of time. With three different values of 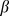: 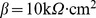 (thick dark line: red online), 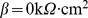 (thick gray line: blue online), 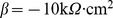 (light gray line: green online). Here 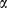 is fixed as 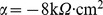. It can be seen that 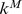 is nearly independent of the parameter 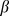.



We can further obtain a theoretical expression of the shunting coefficient. Performing Taylor expansion to the solution of integral form of the modified I&F model to obtain the approximations of EPSP, IPSP, and SSP (see *Methods* for details), then using Eq. (2), we arrive at the shunting coefficient 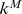 for the modified I&F model
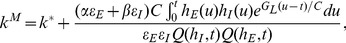
(8)where 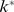 is the shunting coefficient computed in the original I&F model [Eq. (3)]. 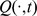 is the same functional as defined in Eq. (3). In Eq. (8), the first term 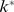 on the right-hand side is independent of the amplitude of EPSP or IPSP, as was shown previously. For the second term, similarly, because 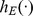 and 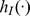 only control the profiles of EPSP and IPSP and are independent of the amplitudes of EPSP and IPSP. Therefore, 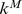 is independent of the amplitudes of EPSP and IPSP and the dendritic integration rule holds for the modified I&F model. Following the same procedure, we can also show that this integration rule holds for the mean potentials. We can further approximate the shunting coefficient 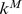 as
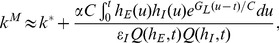
(9)in which 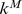 is independent of the parameter 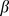. This independence is a consequence of the fact that the magnitude of the inhibitory reversal potential (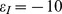 mV) can be viewed as much smaller than that of the excitatory one (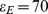 mV) in absolute value. Eq. (9) as an approximation for 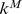 has been verified numerically: Fig. 5 shows that 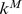 is indeed nearly independent of 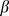. Therefore, we can set 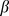 to be zero to obtain a further simplified form of the modified I&F model,

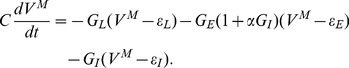
(10)Eq. (10) is our central result and we will refer to this model as a dendritic-integration-rule-based I&F (DIF) model. First, we comment that the above conclusions about the dendritic integration rule remain unchanged for the DIF model. Second, we show that we can incorporate the spatial dependence of the dendritic integration rule using the following parametrization. For a given pair of excitatory and inhibitory inputs, the shunting coefficient 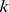 can be measured in the experiment. We can use this measured 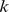 as the value of 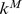 to determine the value of 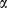 from Eq. (9), namely, we can phenomenologically fit the value of 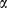 as a function of stimulus locations. Our fitting yields the results which are shown in Fig. 2. The parameter 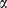 captures the integration effects to the soma arising from both passive cable properties and active conductance properties (ion channels and receptors) of the dendrite [20]. We note that 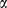 is determined by the shunting coefficient 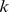 measured with a pair of *concurrent* excitatory and inhibitory inputs. However, even when excitatory and inhibitory inputs are not concurrent, the shunting coefficient 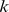 obtained using Eq. (9) is still independent of the amplitudes of EPSP and IPSP because the amplitudes of EPSP and IPSP do not appear in the numerator and denominator. Therefore, the DIF model can be used to study neuronal responses to general stimulus patterns of excitatory and inhibitory inputs.

Finally, we show that the DIF model is consistent with many other experimental observations, some of which have not been obtained by the multi-compartmental model using NEURON software [20].

(1) It has been found in the experiment that SC vanishes when hyperpolarization is induced by somatic current injection 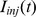 instead of conductance input 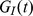 on the dendrite [20]. For this case, the drive can be modeled by 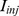 = 
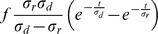
, where 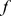 is the magnitude, 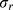 and 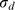 are the rise and decay time constants, respectively [33]. The dynamics of the DIF model becomes
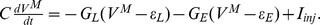



**Figure 6:**
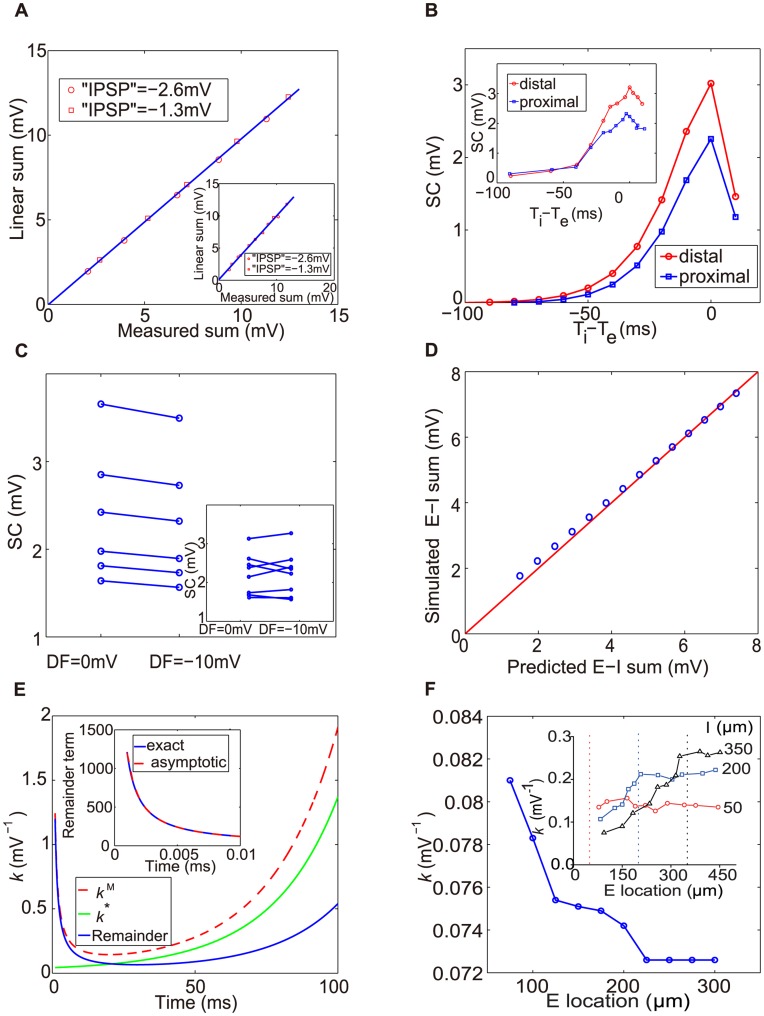
Comparison of DIF model with experiments (A–C), multiple inputs (D), Predictions (E), spatial dependence of 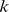 in the I&F model (F). (A) SSP (measured sum) vs. the linear sum between EPSP and direct somatic hyperpolarization. Here, SC is not observed. The inhibition is caused by direct injection of an inhibitory current with amplitude of −2.6 mV (red circle online) and of −1.3 mV (red square online). Inset: experimental measurement (modified from Ref. [20]). (B) SC vs. the relative time delay between IPSP and EPSP. For fixed inhibitory input site, we choose two different input sites for excitation: one corresponds to the distal dendrite (red circle online) and the other the proximal dendrite (blue square online). Inset: experimental measurement (modified from Ref. [20]). (C) SC is not affected by changing the driving force (DF) of IPSP from 0 to −10 mV. Inset: experimental measurement (modified from Ref. [20]). (D) Dendritic integration rule for two excitatory and two inhibitory inputs. The simulated E–I sum represents the SSP obtained from Eq. (12) with a coincident activation of all excitatory and inhibitory inputs, whereas the predicted E–I sum represents the somatic membrane potential obtained from Eq. (11). (E) Comparison between 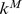 and 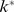 as a function of time. The remainder term is defined as 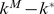 (thick gray, blue online). Inset: The asymptotic behavior 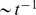 (denoted by “asymptotic”, thick dark dash line, red online) of the remainder term (denoted by “exact”, thick gray, blue online) obtained from Eq. (9). (F) spatial dependence of 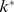 in the I&F model when the inhibitory input site is fixed. Using the spatial dependence of the conductance time constants, we can obtain the result that 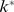 decreases as the distance increases between the excitatory input site and the soma. This is not consistent with the experimental observation (Inset: the same as the inset of Fig. 2).

As verified numerically in *Methods*, the soma response 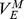 under only excitatory drive can be well approximated by the first-order expansion as 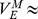 
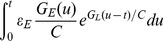
. Therefore, for multiple excitatory inputs, we have the linear summation of EPSPs. This linear summation rule for EPSP has also been found in experiments [36]. We can further obtain 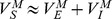: Fig. 6A reproduces the experimental observation (the inset of Fig. 6A) that there is no longer a nonlinear SC term.

(2) It has also been examined how the amplitude of SC is affected by a relative temporal delay between excitatory and inhibitory inputs. In the experiment [20], it was found that (i) the SC amplitude decreases with the length of delay interval between excitation and inhibition and (ii) A larger SC is induced when the excitatory input is located on the distal dendrite than that on the proximal dendrite. The experimental observation (i) can be explained as follows. The shorter the temporal delay between excitation and inhibition, the larger the SC because the amplitude of SC relies on the product between 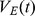 and 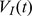. Due to this product form, there is a kink structure for the SC, which can be seen both in the experiment (the inset of Fig. 6B) and in our DIF model (Fig. 6B). The observation (ii) can be understood as follows: A distal excitatory input indicates a larger shunting coefficient 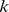, which leads to a larger absolute value of 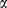 in Eq. (9), therefore, SC is larger for the distal excitatory input. Indeed, the DIF model can capture this time-delay shunting effect successfully as shown in Fig. 6B. The above experimental phenomena have not been addressed by the multi-compartmental model in Ref. [20].

(3) By changing the driving force for IPSP from −10 to 0 mV, it has been found in the experiment [20] that the nonlinear SC term is not affected (the inset of Fig. 6C). In the DIF model, SC can be obtained as 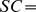 
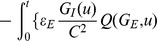
+
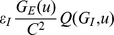
−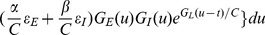. As discussed above, the ratio of the reversal potentials between excitation and inhibition can be viewed as large (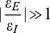), therefore, we have
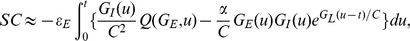
which is independent of 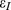. Therefore, a moderate change of the driving force for IPSP in the DIF model would not affect the value of SC. As shown in Fig. 6C, the result of our DIF model agrees with the experimental observation. This independence of the inhibitory driving force shows that the nonlinear term in Eq. (1) is consistent with the notion of shunting inhibition [20]. Using Eq. (2) along with the above equation, we obtain



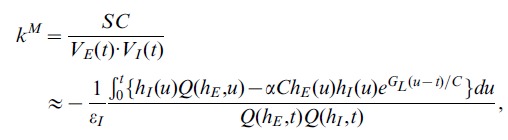
which shows that the shunting coefficient 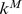 is inversely proportional to 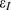 in the DIF model. This relation has also been found by using the multi-compartmental model in Ref. [20].

### DIF Model for Neuronal Network

Note that a pyramidal neuron normally receives a large number of excitatory and inhibitory synaptic inputs [37]. It has been examined by simulation [20] whether the dendritic integration rule obtained from a pair of excitation and inhibition is applicable to multiple excitatory and inhibitory inputs. By using the NEURON software, the following relationship has been found
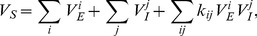
(11)where 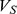 is the SSP with a coincident activation of all excitatory and inhibitory inputs, 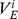 and 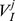 are the individual EPSP and IPSP, respectively. 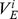 is induced by the 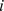
th excitatory input alone and 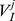 is induced by the 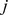
th inhibitory input alone [20]. To account for multiple excitatory and inhibitory inputs and related dendritic integrations, we need to further generalize the DIF model. We propose the following natural extension:
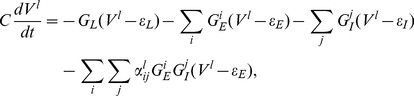
(12)where 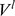 is the membrane potential of the 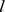
th neuron, 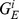 represents the 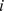
th excitatory conductance input and 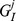 the 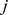
th inhibitory conductance input, 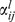 is determined by the shunting coefficient 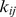 for the pair of the 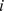
th excitatory and the 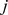
th inhibitory inputs. Using the above model, we study whether the dendritic integration rule has the form of Eq. (11). We tested the case of two excitatory and two inhibitory inputs using several groups of 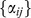, with each 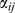 corresponding to the shunting coefficient for each pair of excitation and inhibition. The results are shown in Fig. 6D, which shows that the form of Eq. (11) holds as the dendritic integration rule in the DIF network model [Eq. (12)]. Similar to Eq. (1), Eq. (11) requires that all excitatory and inhibitory inputs occur simultaneously, therefore, one cannot use the formula [Eq. (11)] to calculate the soma responses to the general stimuli of multiple inputs. To study neuronal responses to general inputs of multiple sites, we need to use the DIF network model since it naturally exhibits dendritic integration effects dynamically.

For a given network of 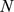 neurons with polysynaptic connectivity, we can use the value of 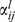 for the 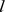
th neuron in Eq. (12) to effectively take into account the dendritic integration effect arising from each pair of synaptic inputs from the 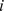
th presynaptic excitatory neuron and the 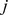
th presynaptic inhibitory neuron. The values of 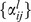 are chosen to model spatial distances of the synaptic locations. Then, we can evolve the dynamics of the neuronal network [Eq. (12)] without explicitly considering the dendritic tree structure for each neuron. Clearly, neither the polysynaptic connectivity nor the value of 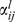 for each pair of synaptic inputs is easy to obtain in current experiments. However, one may numerically study the network dynamics by choosing different values of 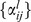, which correspond to different spatial effects of dendritic integration. Our DIF network model might be potentially useful in the numerical study to address the effects arising from dendritic integration in neuronal networks.

## Discussion

In this work, we have proposed a simple DIF neuron model that dynamically incorporates the spatial dependence of the dendritic integration rule by a phenomenological parametrization. Via analytical and numerical methods, we have shown that the DIF model is capable of capturing many experimental observations. Below, we will further discuss properties of this model as well as predictions of this model.

First, we will provide some rationale to the form of the DIF model in Eq. (10). In fact, Eq. (10) can be viewed as a special case of the following equation
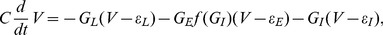
(13)where 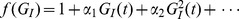. As we have discussed previously, higher order terms of 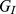 can be ignored since they are too small to have any significant influence on the value of the shunting coefficient 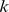. Therefore, Eq. (10) essentially encompasses the major terms which contribute to the dendritic integration [Eq. (1)]. The nonlinear SC term in Eq. (1), which takes the form of the product between excitatory and inhibitory responses, can be understood as follows: the input is through conductances which appear as the multiplication factor of the voltage in Eq. (14). Therefore, linear summation rule for the responses is not necessarily true since the relation between the input and the output response is no longer linear. In other words, the bilinear structure between the conductance and the voltage in I&F-type models gives rise to the bilinear term of the excitatory response 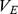 and the inhibitory response 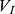 when the excitatory and inhibitory conductance inputs occur simultaneously. Note that, the voltage of neurons in the experiment [20] is substantially below the threshold, therefore, we can use the linear component of the I&F model to model a neuron's dynamical behavior. However, when the voltage is not sufficiently low or when a neuron produces spikes, we may need to use the exponential I&F model [34] or Hodgkin-Huxley-type neuron models to take into account the nonlinear behaviors of a neuron arising from its ion channels. Of course, further experiments should be performed to examine dendritic integration effects in such regimes.

Next, we determine the range of the parameter 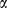 in our DIF model. Notice that, for a fixed excitatory input location, the shunting coefficient 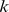 measured in the experiment is between 0.08 and 0.3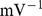 for various inhibitory input locations [20]. From the relation [Eq. (9)] between 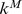 and 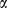, we can determine the range of 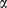: −25 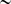 −2 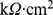. Interestingly, in this range, 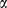 is always negative. As a consequence, the inhibitory conductance input will reduce the effects of excitatory drive, as can be seen from the term 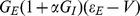 in Eq. (10). In other words, the inhibition is amplified and the neuronal network might be more inhibited than that without dendritic integrations.

### Predictions by the DIF Model

We now turn to the prediction of our DIF model, which can be verified in experiments:

(1) The asymptotic behaviors of 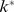 and 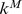 are quite different when the time 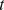 is near zero. As shown in Fig. 6E, 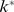 approaches a finite value, whereas 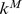 approaches infinity as 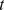 tends to zero. From Eq. (9), the difference between 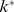 and 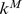 is 
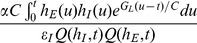
, which we refer to as the remainder term and its asymptotic behavior is 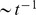 when 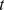 is near zero. In this case, although both the numerator and the denominator of the remainder term are small, the ratio between them can still be very large since the numerator is 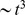 and the denominator is 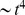 when 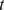 is near zero. By measuring the EPSP, IPSP and SSP at the early time 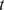 instead of 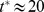
ms in the experiment [20], one could first examine whether the dendritic integration rule [Eq. (1)] still holds. If so, one could further verify whether there is an increase in magnitude for the shunting coefficient 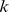 as discussed above. Of course, care should be taken in measuring 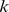 near 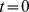 because in this situation the signals are very weak, thus leading to a larger measurement error for 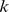.

(2) As discussed previously, the intrinsic origin of the product between EPSP and IPSP in the dendritic integration rule comes from the product between the excitatory and inhibitory conductances. From the DIF model, it is easy to derive the nonlinear relation 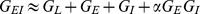, where 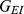 is the conductance when both excitatory and inhibitory inputs are presented. By recording EPSP, IPSP and SSP with high temporal resolution [34], one can construct the corresponding conductances 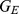, 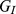 and 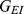 to examine whether such a nonlinear relation holds for any given fixed pair of excitatory and inhibitory inputs.

(3) For synaptic inputs, e.g., inputs are through conductances 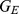 and 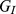, the I&F-type models are no longer linear because they contain the product between the input (conductance) and the response (voltage) as in Eqs. (10) and (14). However, if all the inputs are through direct current injection, the nonlinear SC term in Eq. (1) should vanish as predicted by both the DIF model and the standard I&F model [Eq. (14)].

(4) From the expression of the shunting coefficient 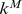 [Eq. (8)], we notice that this dendritic integration rule is not completely attributable to the dendritic properties. The first term on the right-hand-side of Eq. (8) shows that the somatic membrane properties are also responsible for the integration rule. Therefore, if all the conductance inputs are acted on the soma instead of on the dendrite, one should also observe the dendritic integration rule as in Eq. (1). Of course, in this case, the shunting coefficient 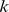 will no longer possess (i) the distal-proximal asymmetry property, and (ii) the dependence of active conductance on the dendrite as observed in the experiment [20] since the input sites of excitation and inhibition are all located at one point (the soma) and the distribution of active conductances (ion channels and receptors) on the dendrite are no longer relevant.

Finally, we point out that the rise and decay time constants of EPSP depend on the input sites for real neurons [38]. Since the shunting coefficient 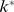 in Eq. (3) is a function of 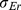, 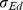, one may wonder whether it is possible to capture the spatial dependence of the shunting coefficient 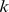 by the I&F model [Eq. (14)] in combination with the input-location dependence of the time constant of EPSP. We first substitute the EPSP's rise and decay time constants measured in the experiment [38] into the fitting function in Ref. [38] to reconstruct the EPSP profile. Then, we use a differential evolution method [39]–-which is a global optimization method–-to search for the best choices of 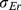 and 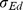 to fit the reconstructed EPSP profile [38]. As shown in Fig. 6F, the value of 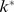 calculated in this manner first decreases as the distance increases between the excitatory input site and the soma, and then saturates at a constant value. The behavior is not consistent with the experimental measurements [20] (the inset of Fig. 6F). In addition, this approach also fails to explain the phenomena mentioned in Fig. 6B.

## Methods

### Conductance-based Integrate-and-fire Model

For the conductance-based I&F neuron model, its dynamics are governed by [33]

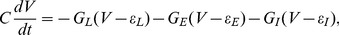
(14)where 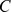 is the membrane capacitance per unit area, 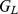 is the leaky conductance, 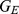 and 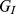 are the excitatory and inhibitory conductances, respectively. 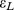 is the resting potential, 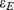 and 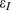 are the excitatory and inhibitory reversal potentials, respectively. The dynamics of conductances 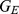 and 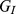 can be described by [40]



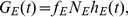
(15)

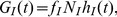
(16)where 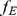 and 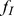 represent the input strength of excitation and inhibition, respectively. 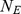 and 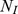 are normalization factors which make 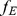 and 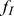 the maxima of 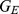 and 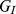, respectively. They are chosen as 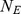 = 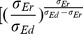
−
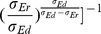
, 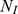 = 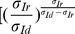
−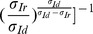. 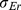 and 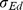 are the rise and decay time constants of the excitatory conductance, respectively. 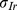 and 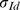 are the rise and decay time constants of the inhibitory conductance, respectively. 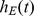 and 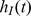 are 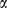-like functions [40] which determine the profiles of EPSP and IPSP, respectively. They are defined as



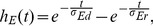
(17)

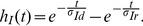
(18)


Since the I&F model only describes the soma dynamics, 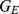 and 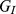 are not synaptic conductances but the effective soma conductances which model the change of the somatic membrane potential due to local synaptic inputs on the dendrite.

In order to be consistent with the setup in the experiment [20], all the voltage values mentioned are chosen as relative voltages with respect to the resting potential. To reproduce the same profiles of EPSP and IPSP as measured in the experiment [20], we choose the time constants as 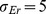
ms, 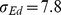
ms, 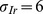
ms, and 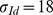
ms. The range of input strength 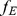 and 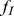 is determined by the range of amplitudes of EPSP and IPSP in the experiment [20]. 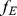 is chosen from 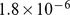 to 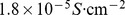 to make the EPSP vary from 1 mV to 10 mV. Similarly, 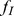 is chosen from 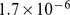 to 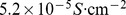 to make IPSP vary from 0.2 mV to 4 mV. The reversal potentials relative to the resting potential are chosen to be the same as those used in the experiment [20]: 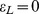
mV, 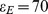
mV, 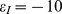
mV, along with commonly used neurophysiological parameters 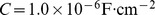, 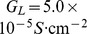 measured in experiments [33]. As shown in Fig. 3, the profiles of EPSP and IPSP measured in the experiment can be well reproduced by the I&F model with the above parameters.

### Analytical and Approximate Solutions to I&F-type Models

Based on the I&F model, the individual EPSP [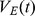] and IPSP [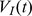] under separate excitatory and inhibitory inputs can be described by
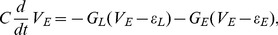
(19)

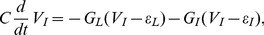
(20)whereas, the SSP [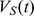] under simultaneous excitatory and inhibitory inputs can be described by



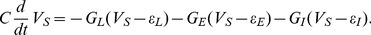
(21)The conductances of excitation and inhibition are given by Eqs. (15) and (16). With notations 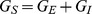 and 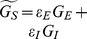, we can obtain analytical solutions to Eqs. (19–21) along with their approximations in integral forms as
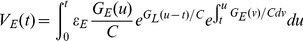
(22a)

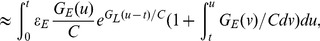
(22b)

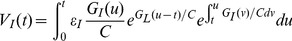
(23a)

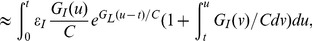
(23b)

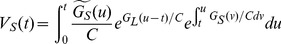
(24a)

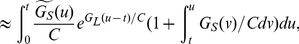
(24b)where approximations are taken with respect to the second order of 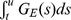, 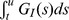, and 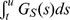 in Taylor expansions. In particular, the soma response 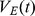 under only the excitatory input can also be approximated by the first-order expansion as 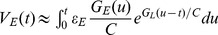.

For the modified I&F model [Eq. (7)], the individual EPSP (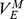) can be obtained by setting the inhibitory input 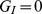 in Eq. (7). For this case, Eq. (7) reduces to Eq. (19). Similarly, for the individual IPSP (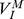) where 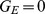, Eq. (7) reduces to Eq. (20). Therefore, we can use Eqs. (23) and (25) as approximations of EPSP (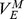) and IPSP (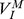). For the SSP (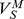), with notations 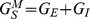
+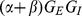, 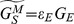
+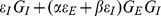, we can obtain the analytical solution to Eq. (7) as
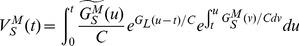
(25)along with the following approximation by performing the second-order Taylor expansion with respect to 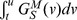:
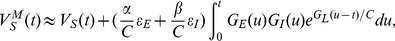
(26)where 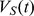 is given by Eq. (24b). All the above approximations have been verified numerically and the relative errors with respect to the analytical solutions are less than 5%.

### Two-port Analysis

A linear relationship between the synaptic current and the membrane potential has been observed in the experiment [41] for fast, non-NMDA input into hippocampal pyramidal neurons. Therefore, we can describe the synaptic currents of excitation and inhibition by 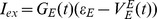 and 
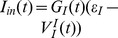
, respectively. Here, 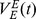 and 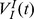 represent the membrane potentials at the sites of excitation and inhibition, respectively. According to linear cable theory [33], the voltage change 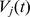 at location 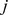 in response to an arbitrary current input 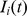 at location 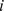 can be expressed as 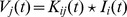, where 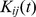 is the impulse response of the system and the symbol “
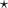
” stands for convolution in time. In particular, for the time-independent case, we can obtain the EPSP at the soma (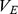) under only the excitatory input as 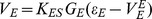, whereas the EPSP at the input site (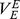) can be obtained as 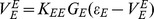. Therefore, we have
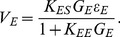
(27)


Similarly, we can obtain the IPSP at the soma (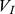) under only the inhibitory input as
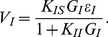
(28)


For simultaneous inputs of excitation and inhibition, the SSP can be expressed as
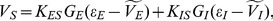
(29)where 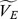 and 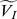 are given by 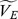 = 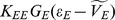
+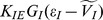 and 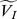 = 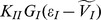
+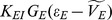, respectively. Solving the above equations, we can obtain



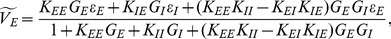
(30)

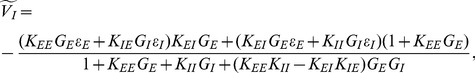
(31)

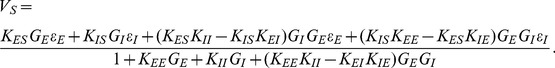
(32)


As pointed out in Refs. [20], [33], the magnitudes of 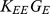, 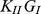, 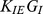, 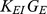 are on the order of 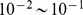 due to small synaptic inputs and thus can be viewed as much smaller than unity. Therefore, we have the following approximations by keeping up to the second-order terms in Taylor expansions
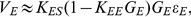
(33)

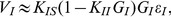
(34)and



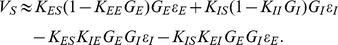
(35)Finally, we can obtain
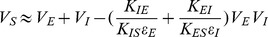
(36)with the shunting coefficient 
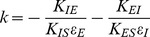
. Eq. (36) has the same form as the dendritic integration rule [Eq. (1)] as observed in the experiment.
